# Sauter-Schwinger effect with a quantum gas

**DOI:** 10.1088/1367-2630/ab3840

**Published:** 2019

**Authors:** A M Piñeiro, D Genkina, Mingwu Lu, I B Spielman

**Affiliations:** Joint Quantum Institute, National Institute of Standards and Technology and University of Maryland, Gaithersburg, MD 20899, United States of America

**Keywords:** quantum gases, Sauter-Schwinger effect, particle creation, quantum simulation

## Abstract

The creation of particle-antiparticle pairs from vacuum by a large electric field is at the core of quantum electrodynamics. Despite the wide acceptance that this phenomenon occurs naturally when electric field strengths exceed *E*_*c*_ ≈ 10^18^ Vm^−1^, it has yet to be experimentally observed due to the limitations imposed by producing electric fields at this scale. The high degree of experimental control present in ultracold atomic systems allow experimentalists to create laboratory analogs to high-field phenomena. Here we emulated massive relativistic particles subject to large electric field strengths, thereby quantum-simulated particle-antiparticle pair creation, and experimentally explored particle creation from ‘the Dirac vacuum’. Data collected from our analog system spans the full parameter regime from low applied field (negligible pair creation) below the Sauter-Schwinger limit, to high field (maximum rate of pair creation) far in excess of the Sauter-Schwinger limit. In our experiment, we perform direct measurements on an analog atomic system and show that this high-field phenomenon is well-characterized by Landau-Zener tunneling, well known in the atomic physics context, and we find full quantitative agreement with theory with no adjustable parameters.

## 1. Introduction

The Sauter-Schwinger effect [1–5] predicts the creation of particle-antiparticle pairs from the quantum vacuum via tunneling when a large electric field is present. This phenomenon arises out of quantum electrodynamics (QED) and the associated pair-creation probability is exponentially suppressed for field strengths below the critical field *E*_c_ ≈ 10^18^ Vm^−1^. Electric fields on this scale are not experimentally accessible; even the largest laboratory fields produced by ultrashort laser pulses [6] fall short, making direct observation of pair creation out of reach of current experiments. Subsequently, analog experiments have been proposed that simulate high-field effects with laboratory accessible energy scales in cold atoms [7–10], graphene [11–16], and other condensed matter systems [12,17–19].

The high degree of experimental control and direct measurement techniques present in ultracold atomic systems allow for quantitative laboratory analogs with quantum gases in an optical lattice. To experimentally probe the Sauter-Schwinger effect with a bosonic quantum gas, we engineered the relativistic 1d Dirac Hamiltonian with *mc*^2^ reduced by 17 orders of magnitude, allowing laboratory scale forces to greatly exceed the Sauter-Schwinger limit. The Dirac picture of particle-antiparticle vacuum and the concept of optical lattice band theory is the foundation of our approach. In this framework, we readily measured pair creation and demonstrated that this high-field phenomenon is well-characterized by Landau-Zener tunneling [20,21] as was discussed in [12,17–19].

In the Dirac vacuum, the enormous required electric field

(1) $$E_{\text{c}}=m_{\text{e}}^{2}c^{3}/\hbar q_{\text{e}}$$

is determined by the particle/antiparticle mass *m*_e_ and charge *q*_e_. For an applied electric field *E*, the pair creation rate is governed only by the dimensionless ratio *E*/*E*_c_, allowing our physical system with very different characteristic scales to be used to realize the underlying phenomenon.

## 2. 1d Dirac Hamiltonian

Our system was well described by the ld Dirac Hamiltonian [ 17,22–25]

(2) $${\hat{H}}_{\text{D}}=c\hat{p}\sigma_{z}+mc^{2}\sigma_{x},$$

where $\hat{p}$ is the momentum operator and σ_*x,y,z*_ are the Pauli operators. Starting with *m* = 0, the ld Dirac Hamiltonian describes particles with velocities ±*c*, that are then coupled with strength *mc*^2^ to give the familiar *ε*(*p*) = ±(*p*^2^ *c*^2^ + *m*^2^*c*^4^)^1/2^ dispersion relation for relativistic particles and antiparticles. At zero momentum, this dispersion has a gap equal to twice the rest mass, at which point the curvature is inversely proportional to the rest mass. The Dirac vacuum consists of occupied states in the lower (antiparticle) band of the Dirac dispersion and vacant states in the upper (particle) band. Vacancies in the antiparticle band represent antiparticles and occupied states in the particle band represent particles. We explored the Sauter-Schwinger limit for pair creation by measuring the probability and rate of ‘pairs’ out of this vacuum as a function of the effective rest mass and applied force.

## 3. Atomic system—1d optical lattice model

We emulated ${\hat{H}}_{\text{D}}$ with the lowest two bands of a ld optical lattice at the edge of the Brillouin zone with the approximate Hamiltonian

(3) $${\hat{H}}_{\text{L}}=\frac{\hbar k_{\text{L}}}{m}p\sigma_{z}+\frac{V}{4}\sigma_{x},$$

giving the pair of relativistic modes shown in figure l. Within this close proximity to the edge of the Brillouin zone, the curvature of the two bands are equal and opposite. The mapping between the scales in ${\hat{H}}_{\text{D}}$ and the analog quantities in ${\hat{H}}_{\text{L}}$ are outlined in table l. The effective rest mass m^*^c^*2^ = *V*/4 is set by the peak-to-valley lattice depth *V* generated by our λ_L_ = l064 nm laser light. The speed of light is replaced by the greatly reduced speed of light $c^{*}=\hbar k_{\text{L}}/m_{\text{Rb}}\approx 4.3$ mm s^−1^ equal to the single photon recoil velocity. The single photon recoil momentum $\hbar k_{\text{L}}=2\pi \hbar /\lambda_{\text{L}}$ specifies the recoil energy $E_{\text{L}}=\hbar^{2}k_{\text{L}}^{2}/2m_{\text{Rb}}=h\times 2.02\;\text{kHz}$. These recoil units set the scale for all physical quantities in our analog system. The effective Compton wavelength λ_C_ = h/m^*^c^*^ = 8λ_L_*E*_L_/*V* is about l0^6^ times larger than that of an electron.

Our simulations consisted of ultracold bosons first prepared in the antiparticle band, then subjected to a constant force, $F_{\text{e}}=q_{\text{e}}E=\hbar \text{d}q/\text{d}t$ modeling an electric field. During the application of this force atoms may transfer from the antiparticle band to the particle band, emulating the pair creation phenomenon. We measured the fraction of atoms transferred to the particle band as a function of the effective rest mass and the applied force. In this manuscript, we begin by using a Bose-Einstein condensate (BEC) to illucidate the connection between Landau-Zener tunneling and pair creation; we then model the Dirac vacuum by uniformily filling the Brillouin Zone of the antiparticle band and observe the predicted rate of pair creation.

## 4. Experimental system

Our experiments began with nearly pure ^87^Rb BECs in the $|F=1,m_{F}=-1\rangle$ internal state, in a crossed optical dipole trap [26] formed at the intersection of two laser beams traveling along **e**_*x*_ and **e**_*y*_, giving trap frequencies (*f*_*x*_, *f*_*y*_, *f*_*z*_) = (44,45,94) Hz. The low density of our N ≈ 10^3^ atom BECs limited unwanted scattering processes in regimes of dynamical instability [27]. The optical lattice potential was formed by a retro-reflected λ = 1064 nm laser beam with a waist of ≈150 *μ*m. Emulated electric forces were applied by spatially displacing the optical dipole beam providing longitudinal confinement (by frequency shifting an acousto-optic modulator). This effectively added a linear contribution to the existent harmonic potential for displacements small compared to the beam waist.

We loaded BECs into the optical lattice by linearly increasing the lattice laser intensity from zero to the final intensity in 300 ms, a time-scale adiabatic with respect to all energy scales. Once the final lattice depth—determined by the laser intensity —was achieved, we applied a force for a time *t*_*F*_. The lattice was calibrated by using Kapitza-Dirac diffraction of the BEC off a pulsed lattice potential [28]. Immediately thereafter, the lattice was linearly ramped off in 1 ms, mapping crystal momentum states to free particles states [29–32]. This process mapped atoms in the antiparticle band to free particle states with momentum between −*k*_L_ and *k*_L_, and mapped atoms in the particle band to states between ±*k*_L_ and ±2*k*_L_. The resulting momentum distribution was absorption imaged after a 15.7 ms time-of-flight.

## 5. Pair creation from a single occupied state

**Figure 2.**
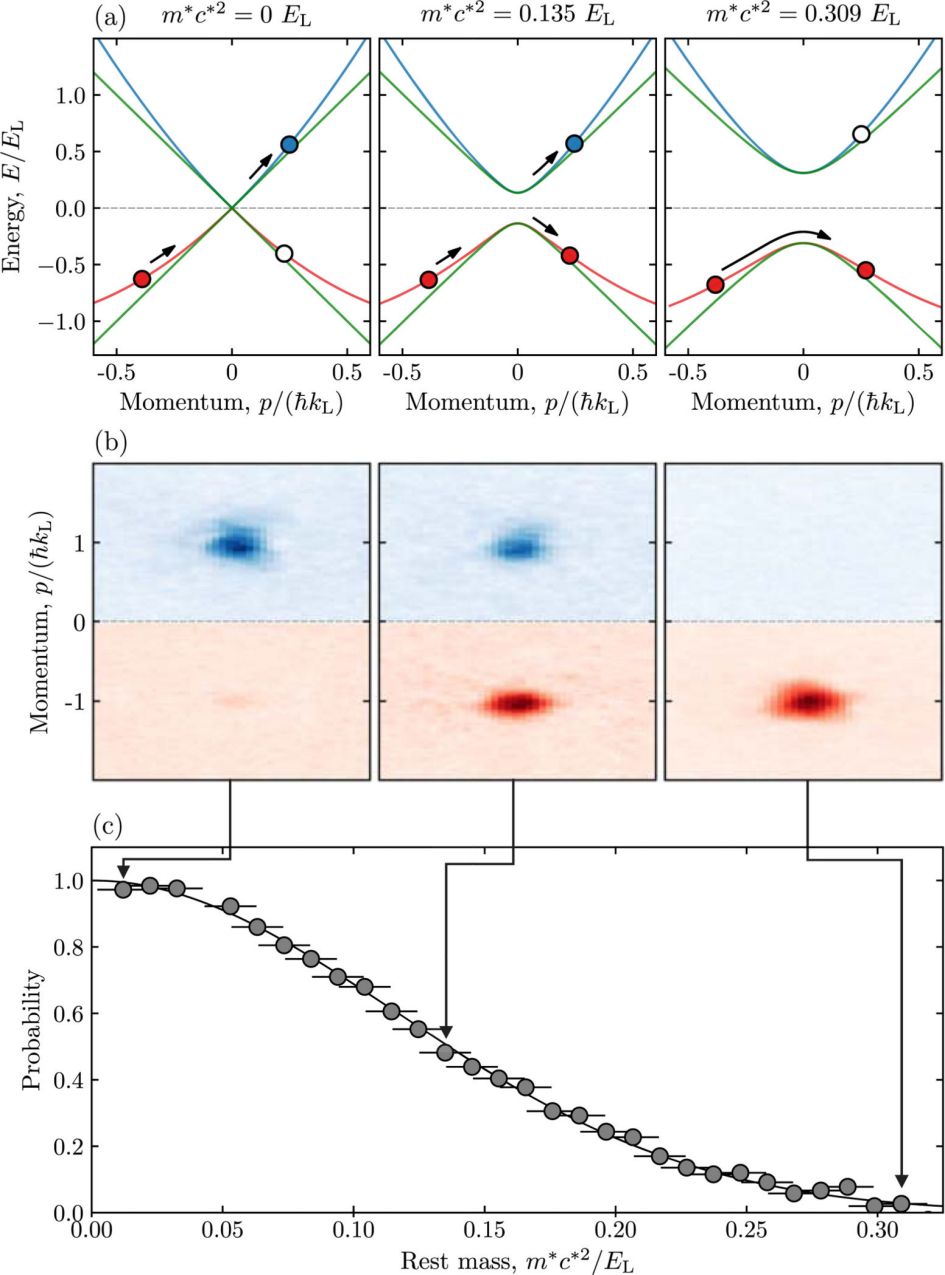
Pair creation from a single state with electric force $F_{\text{e}}=2\hbar k_{\text{L}}/t_{F}$ applied for a time *t*_*F*_ = 3.7 ms. (a) Schematic representation of pair-creation for effective rest mass m^*^c^*2^ (0.012(1), 0.134(9), 0.309(1)) *E*_L_. In each case the green curves denote the Dirac dispersion, and the blue and red curves denote the particle and antiparticle bands respectively. (b) TOF images showing the fractional populations of atoms occupying the particle band (blue tone) and antiparticle band (red tone) for each m^*^. (c) Probability of pair creation as a function of effective rest mass, plotted along with the Landau-Zener model from (3). Statistical uncertainties are shown with typical error bars.

We began with an experiment that is natural in the cold atom setting but is unphysical in high-field physics: we applied an effective electric field and varied the effective rest mass. The result of this changing effective mass is schematically shown in figure 2(a) by an increasing gap at zero momentum. The decreasing probability of pair creation with increasing effective mass anticipated by (1) is schematically illustrated by the filled or partially filled circles. Figure 2(b) shows the distribution of atoms at time *t*_*F*_ selected so that the atoms traversed a full Brillouin zone, i.e. underwent a single Bloch oscillation [33]. Atoms in the top portion of the panel (blue tone) represent particles and atoms in the bottom portion of the panel (red tone) denote filled vacuum states. Figure 2(c) quantifies this effect in terms of the fractional population of a BEC transferred into the particle band, and as expected, the probability of pair creation monotonically decreased with increasing effective rest mass. For small effective rest masses, the atoms almost completely populated the particle band, while for large effective mass the particle band was nearly empty.

The solid curve in figure 2(c) plots the Landau-Zener diabatic transition probability [20,21,23,34–36] given by

(4) $$P_{\text{LZ}}={\text{e}}^{-2\pi \Gamma },\;\text{with}\;\Gamma =\frac{a^{2}}{\hbar \left|\frac{d}{\text{d}t}\Delta E\right|},$$

describing the transit through a crossing with gap *a =* 2m^*^c^*2^ while the massless energy difference *E* (*p*) = 2c^*^p changes as *p* is swept at constant rate d*p/*d*t;* this rate sets the electric force *F*_e_ = *q*_e_*E*. Remarkably in terms of these parameters, the Landau-Zener coefficient is defined by *F*_e_/*F*_c_ = 1/2Γ where *F*_c_ = *q*_e_*E*_c_ is an exact analog of Sauter-Schwinger’s limit of pair creation. This prediction is in near perfect agreement with data in figure 2(c).

**Figure 3.**
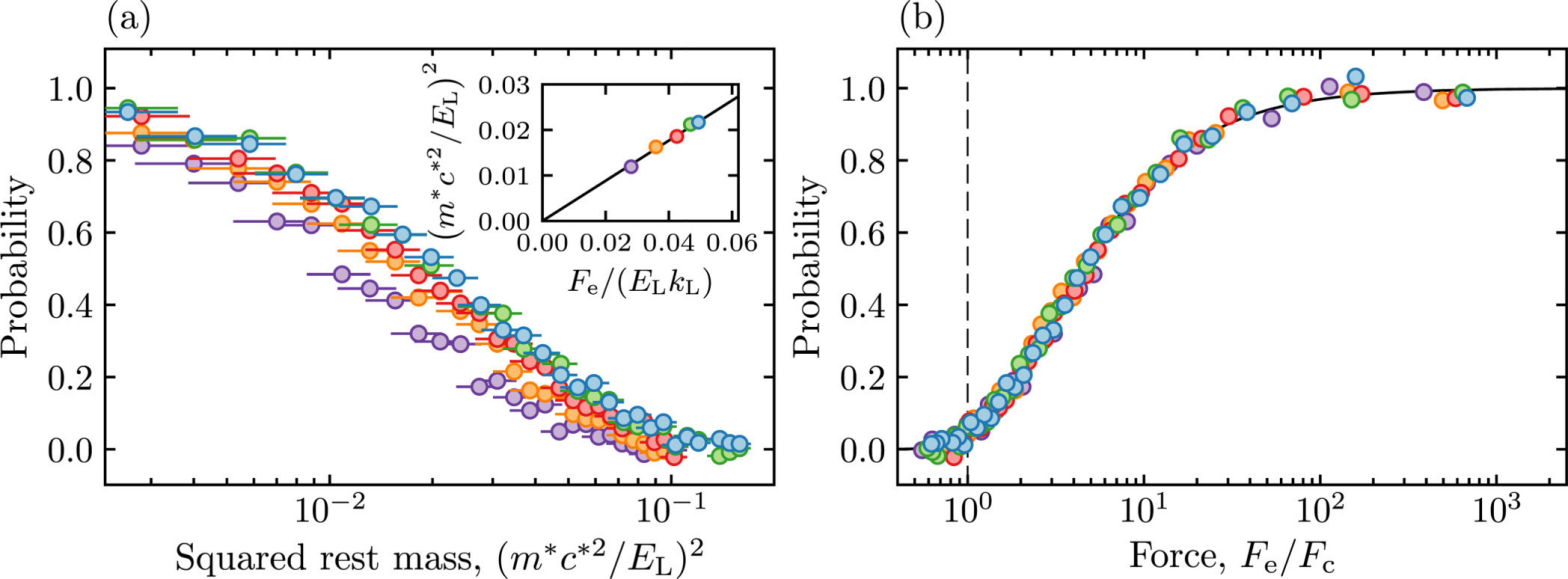
(a) Probability of pair creation as a function of effective rest mass squared for $F_{\text{e}}=2\hbar k_{\text{L}}/t_{F}\;\text{and}\;t_{F}=(3.2,3.4,3.7,4.4,5.6)$ ms, suggesting a common functional form. Statistical uncertainties are shown with typical error bars. The inset quantifies the scaling relationship between these curves in terms of the point where each curve reaches half-max, in agreement with the prediction of (3), shown by the black line. (b) Probability of pair creation plotted as a function of dimensionless force *F*_e_/*F*_c_, showing collapse onto a single curve. The vertical dashed line marks Sauter-Schwinger’s limit where *F*_e_ = *F*_c_, and the solid curve is the prediction of (3).

As suggested by the quadratic dependance on mass in (1), figure 3(a) plots the probability of pair creation as a function of effective rest mass squared for 5 different forces, illustrating their similar behavior. The inset figure confirms the agreement with the Landau-Zener expression by plotting the effective rest mass required to achieve a 50% probability of pair creation as a function of the field strength, and the solid curve shows good agreement with the Landau-Zener prediction. Figure 3(b) displays the same data (circles) now as a function of *F*_e_/*F*_c_ that collapses onto the predicted transition probability (solid curve). This collapse confirms that the physics of pair creation exhibits universal behavior when the field is expressed in units of *F*_c_ as predicted by the Landau-Zener expression. The vertical dashed line marks the ratio *F*/*F*_c_ = 1, the Sauter-Schwinger limit for pair creation; much of our data is in the high-field limit, which is extremely difficult to achieve in other physical contexts. Our data densely samples the critical regime near *F*_c_ and spans the full gamut from vanishing pair creation to nearly complete pair creation. Together these data show the clear connection between the Landau-Zener tunneling of a single quantum state and the Sauter-Schwinger effect. Still, the single occupied state defined by our BEC is far from the Dirac vacuum state.

## 6. Uniformly filled Dirac vacuum

**Figure 4.**
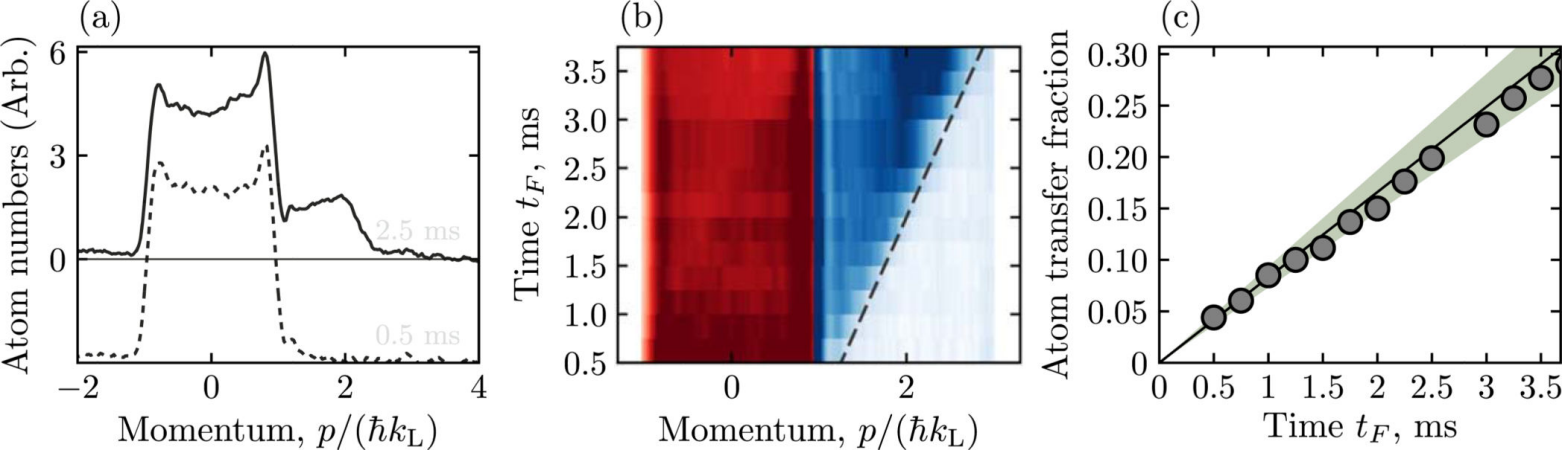
Time dependence of pair creation from the Dirac vacuum using the force $\text{d}q/\text{d}t=2\hbar k_{\text{L}}/t_{2k_{L}}$ derived from the single Bloch oscillation time *t*_2*k_L_*_ = 3.4 ms. (a) Momentum distribution following the application of a force for short (dashed) and long (solid) hold times. (b) Time dependence of pair-creation showing the appearance of and acceleration of particle states and the depletion and deceleration of antiparticle states as a function of time. The red regions and blue regions mark states in the antiparticle and particle bands respectively. The dashed line marks the acceleration expected from the applied force alone. Both the slight wedge shape of the particle-state distribution in (a) and the slight shift from the dashed line in (b) result from the harmonic confining potential. (c) Observed fractional transfer of atoms as a function of time plotted along with the prediction of (3). The green shaded area represents the uncertainty from input parameters.

We therefore created an initial state mimicking the Dirac vacuum in which the negative energy states were occupied with equal probability and the positive energy states were vacant. We prepared this state by first adiabatically loading a BEC into a fairly deep optical lattice (*V* ≈ 3.4*E*_L_, making the lowest bands well separated) and applied a force for 1 s, sufficient for about 300 Bloch oscillations to take place [33]. During this time, crystal momentum changing collisions [27] and dephasing processes uniformly filled the antiparticle band. We then adiabatically reduced the lattice depth in 300 *μs* giving m^*^c^*2^*/E*_L_ = 0.20(1), and proceeded as described in the experimental methods section. Here, pairs were produced at a constant rate for the entire time *t*_*F*_ the force was applied, filling initially vancant states in the particle band while depleting initially occupied states in the antiparticle band. Figure 4(a) shows the atomic momentum distribution following this procedure for small (dashed curve) and larger (solid curve) values of *t*_*F*_. In these data the states in the antiparticle band fall in the pink region, and states in the particle band fall in the blue regions. A uniformly filled initial band would ideally produce a top-hat distribution in the pink region; we confirmed that the observed time-independent peaks in the distribution are consistent with a slight defocus of our imaging system [37–39]. The evolution from the dashed curve to the solid curve clearly shows particles that have been created and then accelerated by *F*_e_. Figure 4(b) summarizes data of this type for variable *t*_*F*_, making clear the appearance of particles already visible in figure 4(a), but also showing a similar reduction of occupation in the antiparticle band. The dashed line marks the anticipated acceleration given by *F*_e_; the slightly reduced observed acceleration results from the harmonic confining potential. Finally, figure 4(c) plots the fractional occupation probability of the particle band, clearly showing the creation of pairs at a constant rate. The line plots the rate, computed from the transition probability in (3), using the force $\text{d}q/\text{d}t=2\hbar k_{\text{L}}/t_{2k_{L}}$ derived from the single Bloch oscillation time *t*_2*k_L_*_ = 3.4 ms for these data. This then is the direct analog of the constant rate of pair creation from vacuum from a uniform electric field.

## 7. Conclusion and outlook

In this experiment, we used a cold atom system to quantitatively probe both the underlying mechanism and the overall phenomena of pair creation as initially conceived of in high-field physics. Our analysis shows that pair creation in an analog atomic system can be equivalently understood as a quantum tunneling process, and our data spans the full parameter regime from low applied field (negligible pair creation) below the Sauter-Schwinger limit, to high field (maximum rate of pair creation) far in excess of the Sauter-Schwinger limit. High-intensity pulsed laser experiments [6,40–43] promise to measure the vacuum nonlinearity and ultimately exceed Sauter-Schwinger’s limit in its original context. Current theory suggests that actual pair creation threshold will be somewhat in excess of *E*_c_ resulting from the Coulomb attraction between electron-position pairs. Future cold atom experiments with repulsively interacting fermions could probe this ‘excitionic’ shift as well, allowing more quantitative comparison with higher order corrections to the threshold field strength. In addition, the pair-creation phenomena occurring in strongly interacting field theories, even absent applied electric fields, may also be realized using mixtures of ultracold bosons and fermions [9,10] and has already been realized using trapped ions [44].

## Figures and Tables

**Figure 1.**
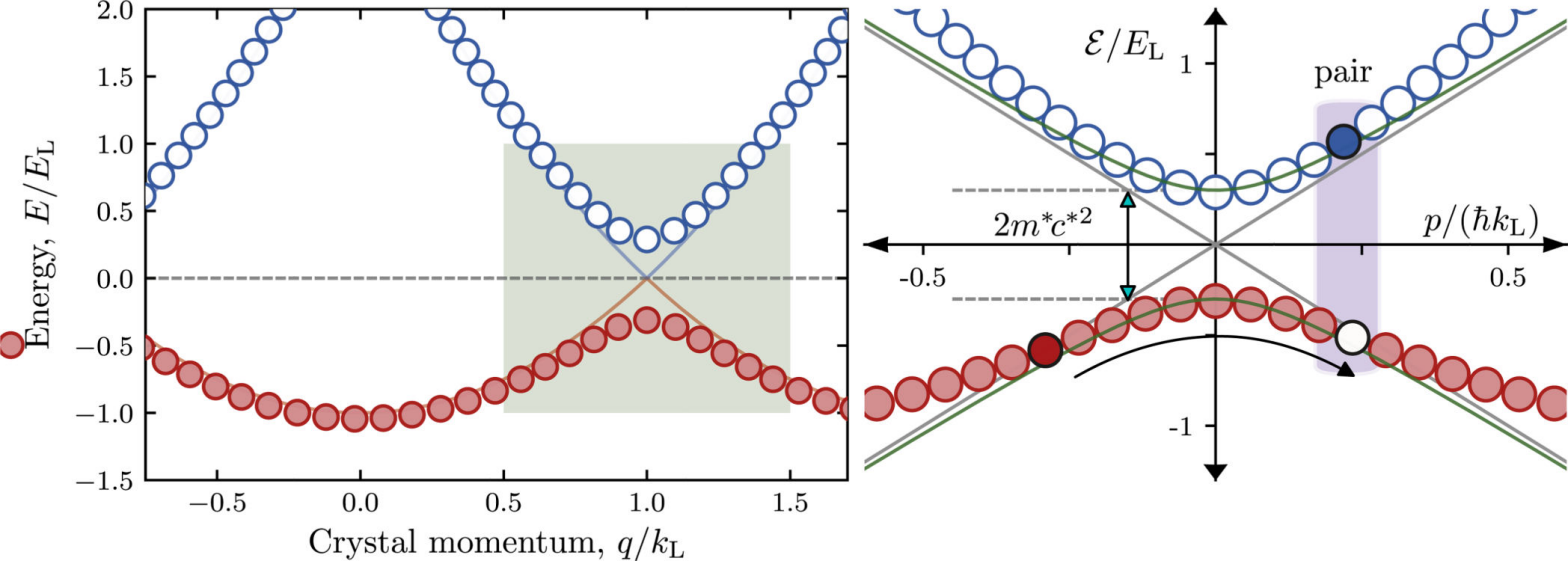
1d Dirac model derived from optical lattice band structure. The left panel shows the lowest two bands of a ld optical lattice and the right panel shows an expanded view at the edge of the Brillouin zone. In both cases, the dispersion of the lower (or ‘antiparticle’) band is denoted by red circles and the upper (or ‘particle’) band is denoted by blue circles. The expanded view reveals the connection to the ld Dirac equation (green shaded region in left panel). The gray lines plot the linear dispersion of massless particles, while the green curves depict the Dirac-dispersion computed from (2). The curved arrow illustrates the process of pair creation where an occupied state in the antiparticle band is converted to an occupied state in the particle band and a vacancy in the antiparticle band.

**Table 1.** Comparison of scales between relativistic electronic systems and our analog atomic system.

	Dirac theory	Analog atomic system
Rest mass	*mc*^2^ ≈ 0.5 MeV	m^*^c^*2^ ≈ 10^−11^ eV
Speed of light	*c* = 299 792 458 m s^−1^	c^*^ = $\hbar k_{\text{L}}$/*m*_Rb_ ≈ 4.3 mm s^−1^
Compton length scale	λ_C_ = *h/mc* ≈ l0^−12^m	$\lambda_{\text{C}}^{*}$ = h/m^*^c^*^ ≈ 10^−6^ m
